# Visualization of Gastrointestinal Bezoar Movement Causing and Releasing Small Bowel Obstruction on Computed Tomography in a Patient With Diabetes Mellitus

**DOI:** 10.7759/cureus.49133

**Published:** 2023-11-20

**Authors:** Hironobu Sugimori, Sho Masaki, Hajime Honjo, Masatoshi Kudo, Tomohiro Watanabe

**Affiliations:** 1 Gastroenterology and Hepatology, Kindai University Faculty of Medicine, Osaka-Sayama, JPN

**Keywords:** lactic acidosis, computed tomography, small bowel obstruction, diabetes mellitus, gastric bezoars

## Abstract

Although delayed gastric emptying promotes gastrointestinal bezoar formation in patients with diabetes mellitus (DM), the association between movement of gastrointestinal bezoars and glycemic status remains unclear. We report a case of small bowel obstruction (SBO) caused by impaction of the migrated gastric bezoar into the small bowel in a patient with DM. Correction of hyperglycemia and lactic acidosis led to normalization of gastrointestinal motility, followed by expulsion of the impacted bezoar and resolution of SBO. This case suggests a link between hyperglycemia, metabolic acidosis, and gastrointestinal motility based on visualization of gastrointestinal bezoar movement in the gastrointestinal tract using computed tomography.

## Introduction

Patients with diabetes mellitus (DM) often display impaired gastrointestinal (GI) motility [1-4]. Such impaired GI motility leads to manifestations of DM-associated GI symptoms including delayed gastric emptying, a risk factor for the formation of gastric bezoars [3,5]. Although migration of gastric bezoars can cause small bowel obstruction (SBO) upon impaction into the small bowel, the association between DM and SBO due to gastric bezoars has been poorly understood [5-7]. In addition, the effects of glycemic status on the movement of GI bezoars causing and releasing SBO have not been reported. Here, we report a DM case with GI bezoars whose movement causing and releasing SBO in response to the glycemic status was visualized on computed tomography (CT).

## Case presentation

A 67-year-old female was admitted for epigastralgia and vomiting. She was diagnosed with type 2 DM and treated with a subcutaneous injection of insulin in combination with oral vildagliptin-metformin hydrochloride. DM control was poor, as indicated by a high hemoglobin A1C (HbA1c) level. The serum glucose level was very high (Table 1). 

**Table 1:** Laboratory Data on Admission

Hematology		Reference Value	Glucose/Ketone Metabolism		Reference Value
White blood cell	10,260 /µL	3,300-8,600	Blood Glucose	618 mg/dL	73-109
Neutrophil	84.9 %	38-77	Hemoglobin A1c	12.8 %	4.9-6.2
Lymphocyte	7.6 %	20.2-53.2	Immunoreactive insulin	5.15 µU/mL	0-18.7
Eosinophil	0.0 %	0.2-4.1	Serum C-peptide immunoreactivity	2.01 ng/mL	0.8-2.5
Basophil	0.1 %	0.2-1.3	Total ketone bodies	1044 µmol/L	0-130
Monocyte	7.4 %	2.7-9.3	Acetoacetic acid	380 µmol/L	0-55
Red blood cell	618x10^4^/µL	386-492 x10^4^	3-hydroxybutyric acid	664 µmol/L	0-85
Hemoglobin	18.4 g/dL	11.6-14.8	Lactic acid	37.6 mg/dL	4 -16
Hematocrit	53.6 %	35.1-44.4	Serology		Reference Value
Platelet	23.2x10^4^/µL	15.8-34.8 x10^4^	C-reactive protein	5.337 mg/dL	0-0.14
Biochemistry		Reference Value	Glutamic acid decarboxylase antibody	Negative	-
Total protein	6.5 mg/dL	6.6-8.1	Urine Analysis		Reference Value
Albumin	3.8 mg/dL	4.1-5.1	Protein	1+	-
Blood urea nitrogen	42 mg/dL	8-20	Sugar	4+	-
Creatinine	0.93 mg/dL	0.46-0.79	Ketone body	4+	-
Total bilirubin	0.8 mg/dL	0.4-1.5	Occult blood	Negative	
Aspartate aminotransferase	14 U/L	13-30	Venous Blood Gas Analysis		Reference Value
Alanine aminotransferase	25 U/L	7-23	pH	7.237	7.35-7.45
Alkaline phosphatase	93 U/L	38-113	pCO_2_	36.5 mmHg	―
Lactate dehydrogenase	220 U/L	124-222	pO_2_	41.2 mmHg	―
γ-glutamyl transpeptidase	34 U/L	9-32	HCO3^-^	15.2 mmol/L	22-26
Creatine phosphokinase	29 U/L	41-153	Base excess	-12.3 mmol/L	-2-+2
Amylase	45 U/L	44-132	Coagulation		Reference Value
Na	135 mmol/L	138-145	Prothrombin time	85.3 %	70-130
K	4.5 mmol/L	3.6-4.8	Activated partial thromboplastin time	34.0 sec	24-39
Cl	98 mmol/L	101-108	Fibrinogen	550 mg/dL	200-400
Ca	8.9 mg/dL	8.8-10.1			
P	6.7 mg/dL	2.7-4.6			

Venous blood gas analyses showed metabolic acidosis. Serum concentrations of lactic acid were markedly elevated whereas serum immunoreactive insulin and serum C-peptide immunoreactivity were normal. Serum levels of total ketone bodies, acetoacetic acid, and 3-hydroxybutyric acid were elevated albeit lower levels as compared with typical cases with diabetic ketoacidosis (DKA) (Table 1) [8]. These data support the diagnosis of lactic acidosis rather than DKA in type 2 DM [8-10]. 

**Figure 1:**
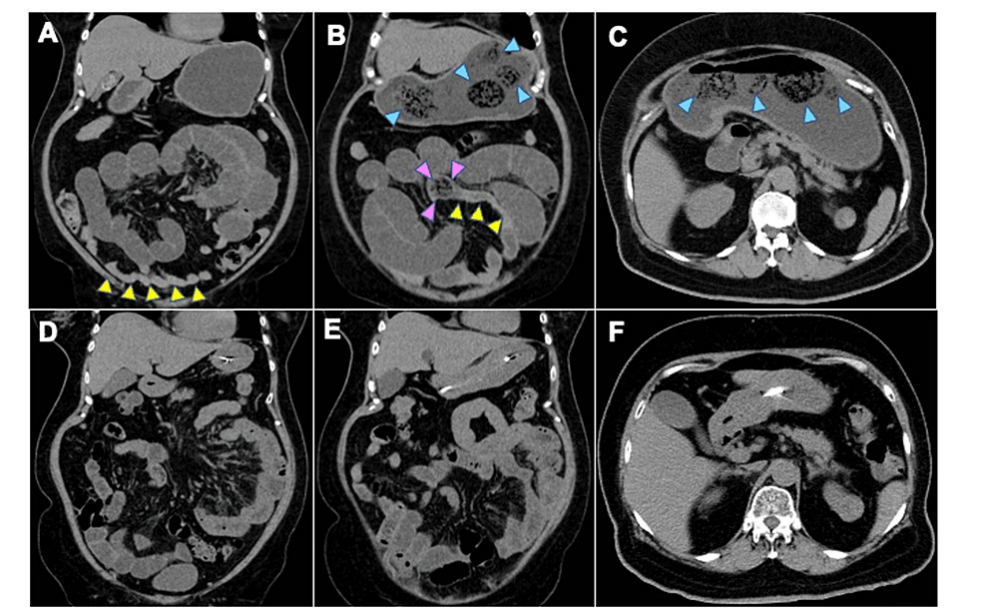
Abdominal computed tomography showing small bowel obstruction due to the impaction of an enterolith. Dilatation of the proximal small bowel loops and collapse of the distal small bowel loops (yellow arrowheads) are observed (A, B). An enterolith (3 cm in diameter; pink arrowheads) is present at the abrupt transition point between the dilated and collapsed small bowels (B). Multiple bezoars (blue arrowheads) are detected in the stomach (B, C). Resolution of the small bowel obstruction was achieved after the treatment (D, E, F), accompanied by the expulsion of the enterolith and gastric bezoars. Neither enteroliths causing small bowel obstruction nor gastric bezoars were detected.

Dilatation of the proximal small bowel loops and collapse of the distal small bowel loops were observed on abdominal CT (Figure 1A).

Additionally, there was an enterolith (3 cm in diameter) with an abrupt transition point between the dilated and collapsed small bowel (Figure 1B). These findings were consistent with those of SBO due to the impaction of the enterolith on the small bowel. Multiple bezoars were observed in the stomach (Figures 1B, 1C). Consistent with previous studies, bezoars and enteroliths displayed well-defined masses with mottled gas patterns on CT [7,11]. Thus, the patient was finally diagnosed with lactic acidosis and SBO, the latter of which was caused by the migration of the gastric bezoar and subsequent impaction into the small bowel.

A nasogastric tube was inserted to decompress the stomach and small intestine. Insulin infusion and fluid therapy were performed according to the protocol for DKA treatment to normalize blood glucose levels [8]. On the day after admission, her symptoms disappeared, accompanied by a marked reduction in serum glucose levels (187 mg/dL) and normalization of lactic acidosis. Follow-up abdominal CT studies verified the resolution of the SBO (Figure 1D). Notably, no enteroliths causing SBO or gastric bezoars were present (Figures 1E, 1F). In addition, dilation of the proximal small bowel loops and collapse of the distal small bowel loops markedly improved (Figures 1D, 1E). These CT findings were consistent with the expulsion of the enterolith and the subsequent resolution of the SBO. Oral intake was initiated, and SBO recurrence was not observed. She was discharged after adjusting the types and doses of insulin as follows: subcutaneous injections of insulin lispro (7 U, 4 U, and 4U prior to breakfast, lunch, and dinner, respectively) in combination with a subcutaneous injection of insulin degludec (2 U).

## Discussion

The most interesting finding in this case is successful CT monitoring of the movement of GI bezoars in relation to glycemic status and metabolic acidosis. The movement of GI bezoars causing and releasing SBO was clearly visualized on CT. SBO due to impaction of the migrated gastric bezoar into the small bowel was observed using CT performed on admission (Figure 1). The following day, resolution of SBO was achieved by expulsion of the impacted bezoar, which was accompanied by the normalization of hyperglycemia and metabolic acidosis (Figure 1). Based on these CT findings, we considered the following link between hyperglycemia, lactic acidosis and SBO: first, the migration of gastric bezoars into the small bowel occurs in a state of normoglycemia or mild hyperglycemia. Second, the impaction of GI bezoars into the small bowel causes SBO via decreased GI motility associated with hyperglycemia because hyperglycemia has been shown to decrease GI motility [12-15]. Finally, dehydration and hypoperfusion induced by both SBO and hyperglycemia lead to the development of lactic acidosis [9,10]. In line with this scenario, normalization of hyperglycemia and metabolic acidosis by fluid and insulin therapy led to the rapid resolution of SBO by expulsion of the impacted enterolith via the recovery of GI motility although the decompressing effects of a nasogastric tube on the GI tract also contributed to SBO resolution. Collectively, the CT findings faithfully reflect the relationship between the movement of bezoars and the degree of hyperglycemia and lactic acidosis; the migration rate of the bezoars was inversely related to the degree of blood glucose levels as well as metabolic acidosis. Having said that, we need to emphasize the roles played by decompression and bowel rest for the rapid improvement of bowel movement.

Delayed gastric emptying is considered a risk factor for the formation of gastric bezoars [5]. Patients with DM often exhibit gastric emptying abnormalities and are recognized as a high-risk group for gastric bezoars although morbidity of gastric bezoars in DM has not been reported [3,5]. SBO, caused by the migration of gastric bezoars into the small bowel, is a major complication of gastric bezoars [5-7]. Here, we report a case with lactic acidosis and SBO due to the migration of a gastric bezoar. Although the presence of DM is a strong risk factor for the formation of gastric bezoars, no case report has described the link between bezoar-associated SBO and DM. In this regard, previous studies showed that hyperglycemia delays the food transition time in the GI tract by decreasing the magnitude of bowel contractions [12-15]. Thus, bezoar-associated SBO can occur in patients with DM, especially when hyperglycemia decreases GI motility. Collectively, this case suggests that the presence of gastric bezoars may be a risk factor for SBO in DM. Therefore, fair glycemic control and careful monitoring of blood glucose levels would be required in patients with bezoars to avoid bezoar-associated SBO. Although Coca-Cola has been reported as effective for dissolving gastric bezoars [5], intake of Coca-Cola was not used in this case due to hyperglycemia.

Based on typical CT findings, i.e., well-defined masses with mottled gas patterns, we considered that masses with various sizes are GI bezoars in this case [7,11]. Although the utility of CT for the diagnosis of GI bezoars is well-established [7,11], neither endoscopic examinations nor surgical resection had been performed in this case. Thus, we cannot completely exclude the possibility that food residues rather than bezoars caused SBO and DKA. Alternatively, spontaneous fragmentation or degradation of GI bezoars might occur by normalization of GI motility during the treatment for SBO because spontaneous resolution of GI bezoars is sometimes observed [5]. Whether SBO was caused by the impaction of bezoars or food residues, this case suggests an association between SBO, hyperglycemia, and metabolic acidosis in type 2 DM. However, we would like to emphasize that CT findings in this case were fully compatible with those of GI bezoars [7,11].

## Conclusions

We report a type 2 DM case in which the movement of GI bezoars causing and releasing SBO on CT was linked to the degree of hyperglycemia and metabolic acidosis. Importantly, hyperglycemia was accompanied by the development of SBO in this case. SBO of this patient was successfully treated with glycemic control and correction of acidosis in addition to decompression by nasogastric tube and bowel rest. Given the high incidence of GI bezoars in patients with DM, the risk of SBO in DM needs to be evaluated in further studies. 
